# Occurrence of waterborne pathogens and antibiotic resistance in water supply systems in a small town in Mozambique

**DOI:** 10.1186/s12866-022-02654-3

**Published:** 2022-10-08

**Authors:** Elisa Taviani, Harold van den Berg, Fernando Nhassengo, Eugenia Nguluve, Jussa Paulo, Olivia Pedro, Giuliana Ferrero

**Affiliations:** 1 Center of Biotechnology, University Eduardo Mondlane, Avenida de Moçambique Km 1.5, Maputo, Mozambique; 2 University of Genova, Corso Europa 16, Genova, Italy; 3 National Institute for Public Health and The Environment (RIVM), WHO Collaborating Centre for Risk Assessment of Pathogens in Food and Water, P.O. Box 1, 3721 MA Bilthoven, The Netherlands; 4 Collins Ltd, Rua Joseph Ki-zerbo, N. 119 Bairro da Sommerschield, Maputo, Mozambique; 5 IHE Delft Institute for Water Education, Westvest 7, 2611 AX Delft, The Netherlands; 6 WASH consulting, Achterom 80, 2611 PS Delft, The Netherlands

## Abstract

**Background:**

. Microbiological quality of drinking water supplied in Moamba, a small town in southern Mozambique, was assessed by collecting and analyzing 91 water sample from 5 sampling sites: raw or inlet water, treated water and 3 household taps along the water distribution system. The presence of *Escherichia coli* as indicator fecal contamination, three bacterial pathogens, *Vibrio cholerae*, *Salmonella* and *Campylobacter* spp., and Cefotaximee resistant *E. coli* as antibiotic resistance determinant, was assessed.

**Results:**

. The results showed fecal contamination in all types of water samples: *E. coli* was found in 100% of inlet water samples, in 21% of treated water samples, and in 22% of tap water samples. No *Salmonella* spp. was detected during the study. The presence of *V. cholerae* was detected in 42% of all water samples tested: 100% of inlet water samples, in 16% of treated water samples, and in 23% household tap water samples. All *V. cholerae* confirmed isolates where genotyped by PCR as non-O1/non-O139; however, 9 isolates showed the presence of the genes encoding for cholera toxin. The presence of *Campylobacter* spp. was detected in 36% of the water samples tested: in 95% of inlet water samples, in 10% of treated water samples and in 23% household tap water samples. Cefotaxime resistant *E. coli* was detected in 63% of inlet water, 16% of treated water, and in 9% of tap water samples, these isolates were also resistant to multiple other antibiotics: ampicillin, streptomycin, tetracycline chloramphenicol. All 70 *V. cholerae* non-O1/non-O139 confirmed isolated were resistant to ampicillin, 51% to streptomycin, 13% to gentamycin, and 1 isolate was resistant to tetracycline; 13% showed a multi-drug resistant profile, being resistant to at least three antibiotics.

**Conclusion:**

. The presence of fecal contamination and pathogens in the water treatment system and household taps in Moamba indicates a health risk for the population. This burden increases by the presence of bacterial pathogens showing multidrug resistance.

**Supplementary information:**

The online version contains supplementary material available at 10.1186/s12866-022-02654-3.

## Background

Universal access to water, the source of life for every human being and for the survival of the planet, has been recognized as a universal human right [1]. Several pathogens such as enteric bacteria, viruses and parasites, are transmitted through consumption or exposure to contaminated water and cause major diseases that represent a global public health problem, particularly for children under the age of five [2, 3]. Despite growing efforts to ensure access to safe water, an estimated 20 million citizens will be exposed to contaminated water by 2030, and waterborne infections and epidemics continue to be a major global public health concern [4]. Contaminated water represents also a possible route of human exposure to antibiotic resistant pathogens of environmental origin [5]. Antibiotic resistance is increasingly a global public health concern leading to millions of deaths due to drug-resistant infection every year, with 700.000 deaths related to antimicrobial resistance are recorded annually [6]. It is estimated that antimicrobial resistant infections may become the leading cause of death globally by 2050 with more than 300 millions (4.500.000 only in Africa) premature deaths expected because of drug resistance [6].

Sub-Saharan Africa is the area most at risk among the Sustainable Development Goals (SDG) regions [2]. Several countries in this region rely on intermittent water supply (IWS), which provide piped water to consumers for less than 24 h per day. The risk of waterborne diseases due to microbial contamination of water in IWS is often high due to the ingress of pathogens in non or low-pressurized pipes through intrusion, back flow, release of particulates, or sloughing of biofilms [7, 8]. Furthermore, the reduced availability of piped water associated with IWS forces households to store water and/or the use alternative unsafe water sources, practices that increase the exposure to contaminated water [9–11].

In order to reduce waterborne diseases, an adequate assessment of the presence of pathogens is essential to implement appropriate water treatment practices [4, 12, 13]. However, there are no universal methods of detection and identification of waterborne pathogens that are applicable to different socio-economic contexts, which makes it difficult to obtain comparable measures and formulate appropriate policies [14, 15]. Current standard methods for monitoring microbial water quality are based on the detection of fecal indicator bacteria (FIBs), such as *Escherichia coli* or *Enterococcus faecalis*, the presence of which indicates fecal contamination of water [16]. However, an inconsistent relationships between FIB and enteric pathogens occurrence in drinking water have been reported in different settings worldwide [17]. Consumption of water free from FIBs have been associated with diarrheal disease outbreaks, likely due to treatment processes that are unable to completely eliminate the pathogens [18, 19]. Moreover, detection of pathogens in water is not part of routine water quality monitoring, and is restricted to research studies or in case of suspected outbreaks [19].

In Mozambique, information on waterborne diseases infections is relatively scarce but confirms that diarrheal diseases are a significant contributor to morbidity and mortality, especially among young children (8–10%) [20–22]. Enteric infections are predominatly caused by Rotavirus [23, pathogenic *E. coli* [24–27*Salmonella* [28, 29*Campylobacter* [30, and *Vibrio cholerae* [31–33]. The latter continue to represent a major public health burden as Mozambique continues on experiencing recurrent annual outbreaks of cholera in different parts of the country, caused by multi-drug resistant (MDR) *V. cholerae*, with incidences ranging from 0 to 211 per 100,000 population and periodically high case-fatality ratios [34–36]. However, little information is available on the contamination of raw and stored waters, mostly limited to the detection of FIB, with few studies detected the presence of waterborne pathogens by molecular methods [37, 38].

In Mozambique, water supply in small towns, where 15% of the country population resides [39, relies on IWS and it is characterized by high levels of leakage, limited hydraulic capacity and short water supply duration (< 12 h) [40]. Van den Berg et al. [41] investigated the effects of operational strategies, such as increased disinfectant dosage, increased supply duration and first-flush, on drinking water quality in an IWS system in a small town of Mozambique. It demonstrated that water in distribution chain is fecally contaminated (*E. coli* indicator).

The aim of this study was to deepen the work conducted by van den Berg et al. [41] by investigating the presence of waterborne pathogens and antimicrobial pathogens in an IWS system in a small town of Mozambique. Standard cultivation methods were coupled with molecular techniques for the detection of *E. coli* as FIB, Extended Spectrum Beta Lactamase *E. coli* (Cefotaxime resistant *E. coli*) as indicator of antimicrobial resistance, and waterborne pathogens: *Vibrio cholerae*, *Salmonella* spp. and *Campylobacter* spp., and their antibiotic resistance profile. The results of this study are relevant for water operators, policy makers and researchers.

## Results

### Microbiological parameters

#### E. coli

*E. coli* was detected in all sample types: in 100% of inlet water (I), in 21% of treated water samples (T0), and in 22% of tap water samples (T1, T2 and T3). *E. coli* counts for I ranged between 26 and 500 CFU/100 mL, with a mean of 185 CFU/100 mL. T0 showed fecal contamination only in May (133 CFU/100 mL), August (37 CFU/100 mL) of 2018 and May 2019 (20 CFU/mL) (Table 1).

**Table 1:** Results of the microbiological analyses: *E. coli* and cefotaxime resistant *E. coli* (CTX) concentrations (CFU/100 mL) and presence/absence of *Vibrio cholerae* (VC) and *Campylobacter* (Camp). Mean, median, minimum and maximum concentrations for each water samples are shown

		WTP inlet (I)					WTP outlet T0					T1					T2					T3				
		*E.coli*	CTX *E.coli*	*VC*	*Camp*	*E.coli*		CTX*E.coli*	VC	*Camp*	*E.coli*		CTX *E.coli*	VC	*Camp*	*E.coli*		CTX *E.coli*	VC	*Camp*	*E.coli*		CTX *E.coli*	VC	*Camp*	
2018	March	505	5.4	+	-	0		0	-	-	4		0	+	-	7		0	-	-	n/a		n/a	n/a	n/a	
	April	414	3.6	+	+	1		0	-	-	0		0	-	-	0		0	-	-	n/a		n/a	n/a	+	
	May	165	3.6	+	+	133		75.7	-	-	0		28.8	-	-	91		30.6	-	-	69		5.4	-	-	
	June	107	0.9	+	+	0		0	-	-	0		0	+	-	0		0	-	-	0		0	-	-	
	July	212	0	+	+	0		0	-	-	3		0	+	-	0		0	-	-	0		0	-	-	
	August	70.2	2.7	+	+	0		0	-	-	0		0	-	-	32		19.8	-	-	0		0	-	-	
	August	53.1	11.7	+	+	37		0.9	+	-	0		0	+	-	11		0	-	+	0		0	-	-	
	September	50.4	0	+	+	0		0	-	-	0		0	-	+	0		0	-	-	13		0	+	-	
	November	28.8	5.4	+	+	0		10.8	-	-	9		0	+	-	0		0	-	+	7		0	+	+	
	December	27.9	0	+	+	0		0	+	-	0		0	-	+	0		0	-	-	0		0	-	-	
2019	February	61.2	13.5	+	+	0		0	-	+	0		0	-	-	0		0	-	+	6		0	-	+	
	March	67.5	7.2	+	+	0		0	+	-	0		0	-	-	0		0	-	-	0		0	+	+	
	April	42.3	0	+	+	0		0	-	-	0		0	+	+	n/a		n/a	n/a	n/a	n/a		n/a	n/a	n/a	
	May	149.4	216	+	+	20		0	-	-	0		0	+	+	0		0	-	-	0		0	+	-	
	June	135.9	1.8	+	+	0		0	-	-	0		0	-	-	100		2.7	-	-	0		0	+	-	
	July	67.5	2.7	+	+	0		0	-	-	0		0	+	-	0		0	+	-	0		0	-	-	
	August	254.7	0	+	+	0		0	-	-	0		0	-	-	0		0	-	-	0		0	-	-	
	September	266.4	0	+	+	0		0	-	+	0		0	+	-	0		0	-	-	0		0	-	-	
	October	261.9	0	+	+	0		0	-	-	0		0	+	-	0		0	-	+	0		0	-	-	
	**N**	**19**	**19**	**19**	**19**	**19**		**19**	**19**	**19**	**19**		**19**	**19**	**19**	**18**		**18**	**18**	**18**	**16**		**16**	**16**	**17**	
	**no. positive samples**	**19**	**12**	**19**	**18**	**4**		**3**	**3**	**2**	**3**		**1**	**10**	**4**	**5**		**3**	**1**	**4**	**4**		**1**	**5**	**4**	
	**% positive samples**	**100**	**63**	**100**	**95**	**21**		**16**	**16**	**11**	**16**		**5**	**58**	**21**	**28**		**17**	**5**	**22**	**25**		**6**	**31**	**24**	
	Mean	185	14			9		1			0		1			12		3			4		0			
	Std Dev	162	49			31		7			0		7			31		8			17		1			
	Min	26	0			0		0			0		0			0		0			0		0			
	Max	500	216			133		29			0		29			100		30			69		5			

The T1 tap sampling point showed *E. coli* in 3 out of 19 samples with counts less than 10 per 100 mL. At tap T2 *E. coli* was detected in 5 out of 18 samples with *E. coli* concentrations ranging between 11 and 100 CFU/100 mL in August 2018 and June 2019, respectively. In water collected at T3 *E. coli* was detected in 4 out of 16 samples, with concentrations ranging between 6 and 69 CFU/100 mL. In May 2018, all five locations were positive for *E. coli* and/or Cefotaxime resistant *E. coli* (Table 1).

#### Cefotaxime resistant E. coli

Cefotaxime resistant *E. coli* counts were reported in all sample’s types: in 63% (I), 16% (T0), and 9% (T1, T2 and T3). Counts for I ranged between 1 and 216 CFU/100 mL, with a mean of 14 CFU/100 mL (Table 1). Presence of Cefotaxime resistant *E. coli* was confirmed in treated water T0 in May (76 CFU/100 mL), August (1 CFU/100 mL) and November (11 CFU/100mL) of 2018. As for the taps, counts were positive for T1 only in May 2018 (29 CFU/100mL), for T2 in 3 out of 18 samples with concentrations ranging between 3 and 31 CFU/100 mL in May, August 2018 and June 2019, and for T3 in May 2018 (5 CFU/100 mL) (Table 1). Overall a significant correlation with the indicator in water samples was observed (r = 0.6, p-value = 3.945e-10). In May 2018, counts were high for all sample’s types except for T1 water where Cefotaxime resistant *E. coli* was detected but not the indicator (Table 1).

#### Vibrio cholerae

*V. cholerae* was detected in 42% of all water samples tested: 100% (I), 11% (T0), and in 23% (T1, T2 and T3) (Table 1). All *V. cholerae* confirmed isolates where genotyped as non-O1/non-O139 by PCR. In four inlet water samples from September through December 2018 and August 2019 *V. cholerae* non-O1/non-O139 was isolated with the genetic potential of producing cholera toxin (c*tx*^*+*^*).* All other *V. cholerae* non-O1/non-O139 isolates were CTX negative.

#### Salmonella

*Salmonella* spp. was not detected in any of the samples.

#### Campylobacter

*Campylobacter* spp was detected in 36% of the water samples tested (n = 29): 95% (I), 10% (T0) on February and April 2019, and 23% (T1, T2 and T3) on April 2018, from August 2018 through May 2019 and in August 2019 (Table 1).

Table 1.

## Antibiotic resistance

Selected Cefotaxime resistant *E. coli* confirmed isolates were tested for susceptibility to 12 antibiotics. All 15 strains tested showed resistance to multiple antibiotics. As expected all isolates were resistant to Cefotaxime, all were also resistant to ampicillin. Additionally, 10 isolates were resistant to streptomycin, 7 to tetracycline and 1 isolate was also resistant to chloramphenicol (Table 2). Isolates resistant to at least one other antibiotic were detected in all types of water samples tested. Ten isolates (62%) showed a MDR profile, being resistant to at least three antibiotics, five of these were isolated from I, two from T0, one from T1 and two from T2.

**Table 2:** Number of isolates, isolation source and antibiotic resistance pattern of *E. coli* and *V. cholerae*

	*Source*	*No. of isolates*	*Antibiotic resistance*
E. *coli*	Total	15	
	Inlet WTP	4	CTX, AMP
		4	CTX, AMP, TET, STR
		1	CTX, AMP, TET, STR, CHL
	Outlet WTP	1	CTX, AMP
		1	CTX, AMP, TET, STR
		1	CTX, AMP, STR
	T1	1	CTX, AMP, TET, STR
	T2	2	CTX, AMP, STR

All 70 *V. cholerae* non-O1/non-O139 confirmed isolated were tested for susceptibility to 11 antibiotics. Of these, 69 isolates were resistant to ampicillin, 35 (51%) were resistant to streptomycin, 9 (13%) were resistant to gentamycin, and 1 isolate was resistant to tetracycline (Table 2). Nine isolates (13%) showed a MDR profile, being resistant to at least three antibiotics, and of these 5 isolates originated from I, 2 from T1 and 1 from each T1 and T3.

Table 2.

## Physico-chemical parameters

**Fig. 1:**
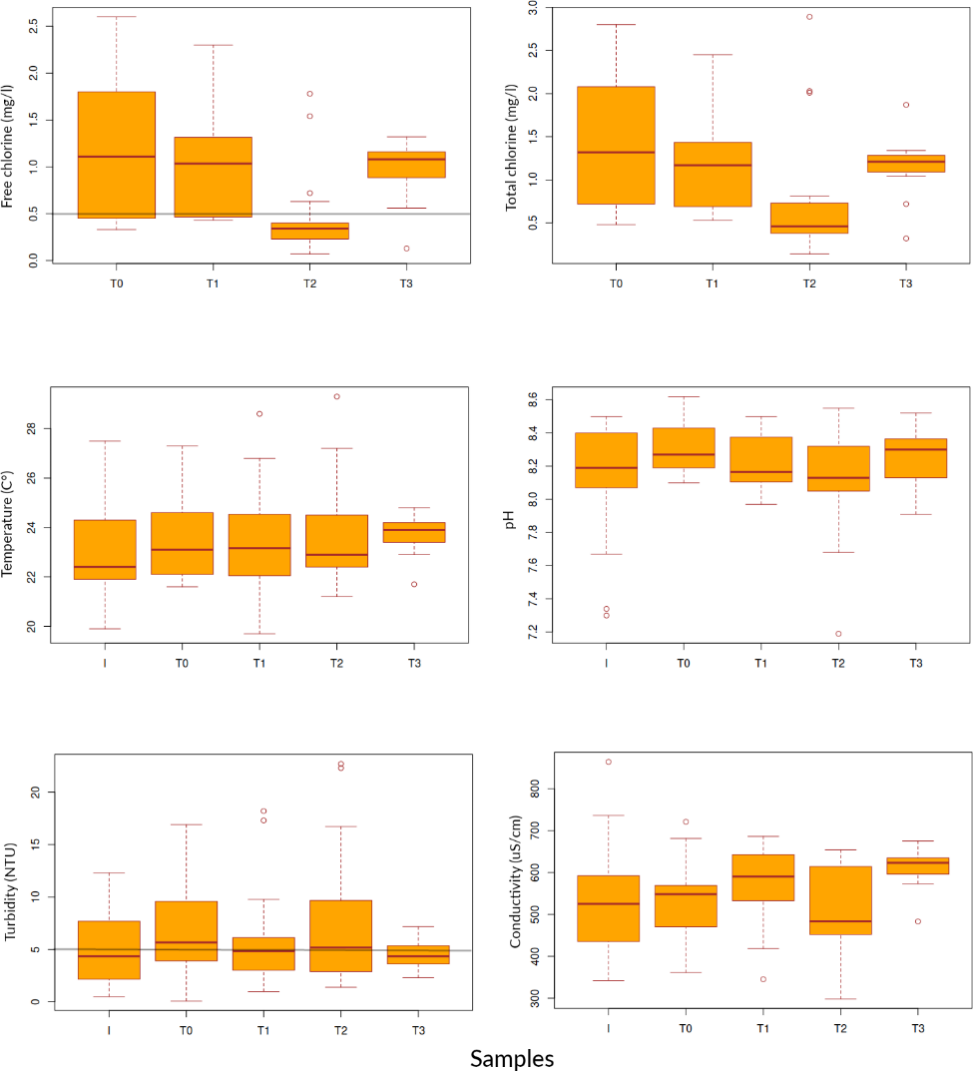
Map of the distribution network of Moamba and location of the WTP (T0) and sampling points in Cimento (T1), Matadouro (T2) and Bairro Sul (T3)

Free chlorine levels varied greatly among sample type. The highest mean value of chlorine was 1.2 mg/L detected at the outlet of the WTP (Fig. 1, Supplementary Table 4). Free chlorine levels at T0 ranged between 0.3 and 2.6 mg/L. In household tap water the level of free chlorine ranged between 0.1 mg/L at T1 in April 2019 to 2.3 mg/L at T2 in April 2018. T1 household water had the lowest mean values of free chlorine (Fig. 1, Supplementary Table 4). In 7% of all the samples taken at household taps (n = 44) free chlorine was less than 0.2 mg/L, in 57% had values comprised between 0.2 and 1 mg/L, and in 36% of the samples the free chlorine exceeded 1 mg/L (Fig. 1, Supplementary Table 4). Differences observed between free chlorine values detected at T1 and T0, T2 and T3 were significant at the 95% interval (p < 0.05).

**Fig. 2:**
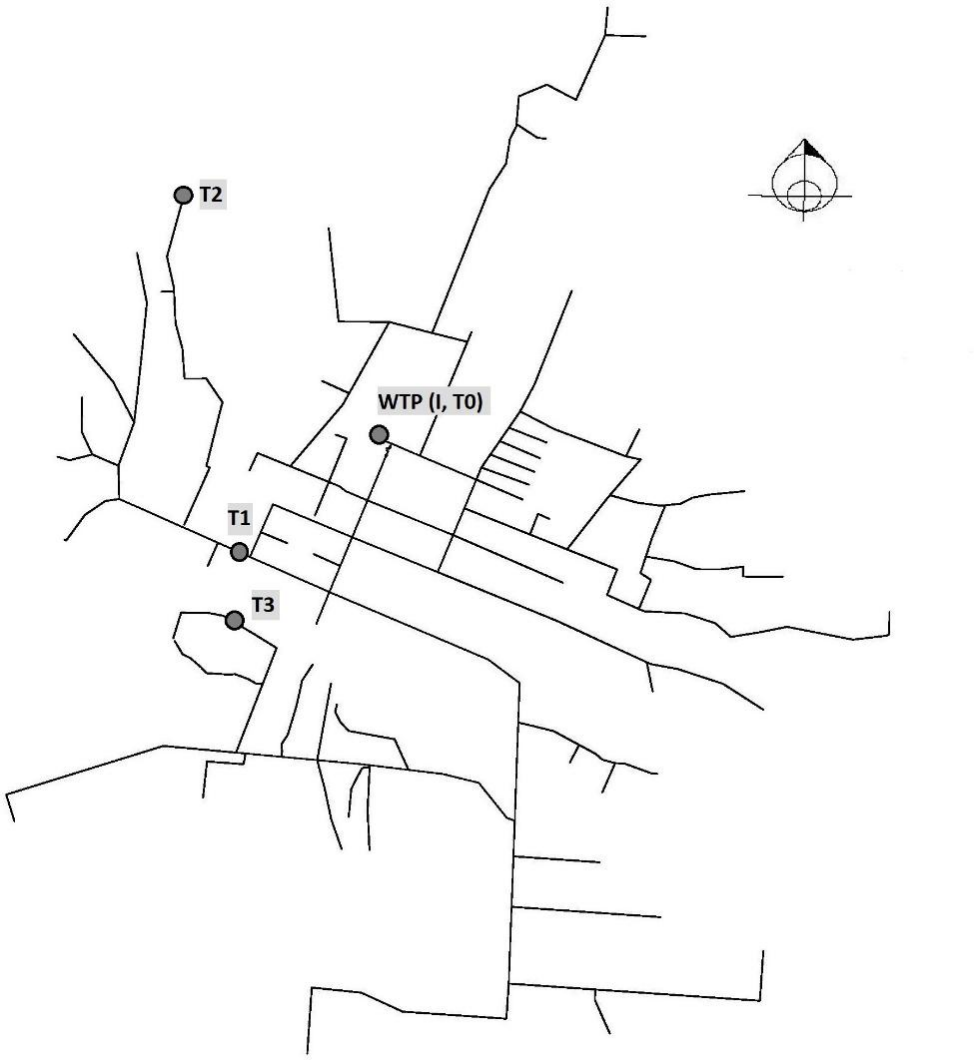
Box-Whiskerplots of physico-chemical parameters of the water collected at sampling points. Grey lines show legal requirements for free chlorine (0.5 mg/l) and turbidity (5 NTU). The box represents the median and quartiles, the whiskers show the 95%-interval and dots are outliers

Turbidity of the Inlet water ranged between 0.5 and 12.3 NTU (Fig. 2, Supplementary Table 4). For T0 water turbidity ranged between 0.1 and 10.3 NTU. Turbidity values recorded at household taps ranged from a minimum of 0.8 NTU to a maximum of 22.7 NTU. 93% of the total number of tap water samples (n = 44) analyzed were greater than 1.0 NTU of which 45% did not comply with the national standards of 5.0 NTU. The highest turbidity level at tap was recorded in March 2018. Statistically, the differences observed were not significant at 95% interval (p < 0.05).

The average temperature of the water remained stable at around 23℃ at all sampling locations throughout the study period. The average pH of the water ranged between 8.1 and 8.3, values fell within the range of legal requirement for drinking water (pH 6.5–8.5). Conductivity of the treated and household tap water ranged between 298 µS/cm and 721 µS/cm, within the range of 50 − 2,000 as legal requirements, with average values above 500 µS/cm (Fig. 2, Supplementary Table 4).

Figure 2.

## Discussion

During this study, the level of fecal contamination of the Inkomati river water (mean value of 185 CFU/100 mL) was lower than levels reported for surface water in the neighboring Limpopo Province in South Africa (mean values of 0.3 to 1.4 × 10^4^ CFU/100 mL) [42, and lower than three rivers in Ecuador (128 to 1248 MPN/100 mL) [43]. *E. coli* counts were in average higher in the dry season (April-October) than in the wet season (November-March) indicating that lower level of river water affects the concentration of microbes.

When considering the removal of fecal contamination at the Moamba WTP, we recorded complete removal of *E. coli* in 79% of the samples. Samples showing *E. coli* contamination were collected in April, May and August of 2018 and June of 2019, and showed 2.62, 0.09, 0.16 and 0.33 log_10_ removal, respectively. In 95% of tap water samples the turbidity was greater than WHO level (1.0 NTU) to guarantee an effective disinfection process [16] (WHO 2017). High turbidity in filtered water is associated with poor removal of pathogens, sloughing of biofilms and ingress of contaminants through broken pipes [16].

Removal efficiency of fecal contamination did not correlate significantly with any of the physico-chemical parameters assessed. Our findings reported re-contamination during distribution at all three household taps, where 23% of water samples exceeded national standards for potable water parameters for *E. coli*. Fecal contamination monitoring of a IWS system in India reported 32% of samples exceeding WHO drinking water quality guidelines [7]. Water collected from household taps was generally free of *E. coli* contamination as 77% of the samples consisted of non-detects. Prevalence of household tap samples contaminated with *E. coli* were 16%, 28% and 25% for Cimento, Matadouro and Bairro Sul, respectively. These values are in line with those reported in another study conducted on the same WTP [41]. Similar values of *E. coli* contamination were reported in Maputo tap water (23% of samples) [38]. Also, the prevalence of fecal contamination observed in our study reflects the variability observed in other IWS distribution networks [7, 8, 44].

Limitations of the fecal indicator paradigm have long been reported with the inconsistent relationships between FIB occurrence, enteric pathogens, and associated health risks [45–48]. The absence of *E. coli* does not eliminate the risk of the water being contaminated by enteric pathogens that may show a higher resistance to disinfection and the ability to persist in the distribution network in biofilms [4, 7]. In our study we have detected the presence of pathogens such as *Vibrio cholerae* and *Campylobacter* throughout the water supply in Moamba even when *E. coli* was not detected. 11% of the treated water samples were positive for *V. cholerae* and *Campylobacter*, while the two pathogens were detected in 18% and 15% of tap water samples, respectively, in absence of *E. coli*. *V. cholerae* survives better in estuarine waters than *E. coli* resulting in poor correlation of *V. cholerae* levels with fecal coliform concentrations in estuarine waters [49]. Also, *V. cholerae* non-O1 is a natural inhabitant of waters and therefore it may have a greater fitness in water than the enteric commensal *E. coli*. In a recent study monitoring treated water quality, *V. cholerae* abundance was higher compared to *E. coli* after three days [50]. The extended survival of *V. cholerae* in treated water may have serious public health implications.

*V. cholerae* non-O1/non-O139 was present in 74% of samples from Inkomati river. This pathogen has been widely reported in surface water in Mozambique and worldwide [51–53]. Nine *V. cholerae* non-O1/non-O139 isolates were positive for the presence of the *ctxAB* genes encoding the cholera toxin, the virulence factor causing the severe diarrhea associated with cholera disease. These isolates originated from inlet (5), treated water (1) and tap water (3) samples. Although rare in the environment, the detection of *ctx* genetic determinant in *V. cholerae* non-O1/non-O139 has been reported in several countries [54, 55, including Mozambique [56]. The presence of this microorganism has been linked to cases of diarrheal diseases, representing a risk for the population consuming the water [57].

Detection of *Campylobacter* in 10% of treated water samples and in 19% household tap water samples further suggested environmental contamination and persistence of pathogens along the Moamba WTS distribution network. The contamination of water by *Campylobacter* can be linked to the presence of a major hatchery and the widespread smallholder family poultry producers in Moamba district [58].

*Campylobacter* detection did not correlate with the occurrence of *E. coli* as indicator of faecal contamination in treated and household tap water samples. In other studies the presence of *Campylobacter* spp. showed a lower correlation with fecal indicators respect to other pathogens such as Cryptosporidium/Giardia, pathogenic *E. coli*, and *Salmonella* spp [59].

Antibiotic resistant bacteria have been increasingly reported globally, not only restricted to clinical settings but also recovered from environmental samples, especially water. The pandemic diffusion of ESBL-producing Gram-negative bacteria in drinking water distribution systems is a major health concern, affecting mostly low-income countries in Asia and Africa [38, 60–63]. In our study, cefotaxime resistant *E. coli*, and *V. cholerae* strains resistant to several class of antibiotics were detected in inlet, treated and household water samples. The high prevalence (62%) of MDR *E. coli*, indicated that different classes of antibiotics are being co-selected with β-lactam resistance in the aquatic environment. A much lower incidence (10.7%) of ESBL *E. coli* was reported in tap water of Maputo [38]. Also, 13% of *V. cholerae* non-O1/O139 isolates showed a MDR profile. Our results confirmed the rapid dissemination of AMR in environments that are not directly affected from major clinical inference, implying an overuse and misuse of antibiotics in local communities [64].

## Conclusion

From our finding we can conclude that the detection and monitoring of major microbial pathogens at different points of the drinking water treatment process and distribution network is crucial for water quality management, especially in IWS where non- or low pressurized pipes permit re-contamination of treated water. A contaminated water distribution system may act as source of waterborne pathogens and a mean for spreading them between communities. On the other hand, in settings like the ones surveyed in our study where low level of sanitation and hygiene allow for circulation of FIB and pathogens between humans, animals and household environment, measurement of standard tap drinking water quality alone may not be sufficient to accurately predict the safety and health implications associate with its consumption. New practices are needed to support monitoring approaches that go beyond the routine measurement of *E. coli* or FIB as mean to assess drinking water quality.

## Methods

### Study area

- This study was conducted in Moamba, a town located in the southern Maputo province of Mozambique. Moamba District has an area of 4,628 square km, and the town has a population of 24,650 inhabitants [65]. Since 2013 the town is supplied by an IWS system with the capacity of 3,000 m³/day. The source for the production of drinking water is the Incomáti river. Water is abstracted 3.5 km from the water treatment plant (WTP) and subjected to coagulation-flocculation based on dosing of aluminium sulphate, rapid sand filtration by six pressure filters with a capacity of 40 m³/hour each, and disinfection by dosing chlorine solution with a calculated dose of 1.8 mg Cl_2_/L^41^.
- The system supplies water to all but three neighborhoods of Moamba, reaching 83% of the population through a distribution network with a total length of 45 km with approximately 3,336 connections. The WTP is operates in two cycles (morning and afternoon) and disinfected water is stored in a 500 m [3] reservoir and 150 m [3] water tower before being distributed via the network [41].

## Sampling locations

Samples were collected from the intake source water for drinking water production (inlet, I), treated water at WTP (outlet, T0), drinking water from 3 taps in different neighborhoods of Moamba: Cimento (T1), Matadouro (T2) and Barrio Sul (T3). Sampling locations had a piping distance from the WTP of 800 m, 2,200 m and 1,863 m for T1, T2 and T3, respectively (Fig. 1).

Figure 1.

Nineteen sampling rounds were carried out on a monthly basis between March 2018 and October 2019 during both the dry (April 2018 - October 2018 and April 2019 - September 2019) and wet seasons (March 2018 and November 2018 - March 2019). A total of 91 water samples were collected at the 5 sampling sites. For sampling trips in March and April 2019 it was not possible to collect water from household T3, and in April 2019 it was not possible to collect water from households T2 and T3, because residents reported lack of water at these sites.

## Water samples collection

Water samples were collected in the morning supply cycle, between 10 and 11 AM. For microbiological and physico-chemical analyses, 500 mL water samples were aseptically collected in sterile collection bottles previously cleaned with detergent and sterilized by autoclaving for 20 min at 121 °C prior to use. Collection bottles were supplemented with thiosulfate tablets (Starplex Scientific Inc, USA) to inactivate available chlorine.

Water samples of inlet and outlet of the WTP and tap water were collected after flushing the water for 2 min and kept in a cooler during transport to Center for Biotechnology laboratory for analysis. Samples were processed on the same day, typically within 2–4 h of collection.

## Microbiological parameters

Enumeration of *E. coli* and cefotaxime resistant *E. coli* as well as detection of the pathogens *Vibrio cholerae*, *Salmonella* spp. and *Campylobacter* spp. was carried out in all water samples (I, T0, T1, T2, T3) by testing different volumes per parameters and per sample location (Supplementary Table 1).

Volumes ranging between 10 mL to 100 mL were filtered through a 0.47 μm polycarbonate membrane and incubated in plates or 50 mL of enrichment selective media. Volumes of 0.1 mL and 1 mL were either added to sterile PBS and filtered or added directly to 10 and 9 mL of specific enrichment selective media, respectively (Supplementary Table 1).

### E. coli

Enumeration of total *E. coli* was done according to ISO 9308-1 standard method. Different volumes (Supplementary Table 1) were filtered and membranes were placed on Tryptone Bile X-glucuronide (TBX) (Merck KGaA, USA) plates and incubated overnight at 35–37 °C. After incubation, plates were checked for growth and presumptive *E. coli* β-glucuronidase-positive (blue/green) colonies in each plate containing less than 300 were counted as colony forming units (CFU).

### Cefotaxime resistant E. coli

Different volumes (Supplementary Table 1) were filtered and membranes were placed onto TBX plates supplemented with cefotaxime (CTX) (4ug/mL) and incubated overnight at 35–37 °C. After incubation, plates were checked for growth and presumptive *E. coli* β-glucuronidase-positive (blue/green) colonies in each plate containing less than 300 were counted as CFU. At least 5 colonies were picked and stored in glycerol at -80℃ for species confirmation by PCR and antibiotic resistance profile.

### Vibrio cholerae

*V. cholerae* was detected as described by Huq et al. [66]. After filtration, membranes were incubated in Alkaline Peptone Water (APW). The enrichment broth was incubated overnight at 35–37 °C, followed by plating onto thiosulfate citrate bile salt sucrose (TCBS) agar (BD, USA) and overnight incubation at 35–37 °C. Plates were checked for growth of presumptive *V. cholerae* (yellow, with a diameter of 2–3 mm) and at least 5 presumptive *V. cholerae* colonies were picked, plated onto Luria Bertani (LB) agar, incubated over night at 35–37 °C and then stored in glycerol at -80℃ for molecular analysis and antibiotic susceptibility.

#### Salmonella

*Salmonella* spp. detection was carried out according to the ISO 19,250 standard method. Membranes were transferred to Buffered Peptone Water for non-selective enrichment during overnight incubation at 35–37 °C. The following day 0.1 mL of enrichment was added to 10 mL of Rappaport-Vassiliadis soya peptone broth for selective enrichment for 24 h at 41.5 °C. Samples exhibiting growth were plated onto Xylose Lysine Deoxycholate (XLD) agar and incubated 24 h at 35–37 °C. Tubes not exhibiting growth were re-incubated for 24 h at 35–37 °C and then checked for exhibiting growth. XLD plates were checked for growth and presumptive *Salmonella* colonies were subcultured onto Brilliant Green (BG) agar plates and incubated for 24 h at 35–37 °C for further confirmation. At least 5 presumptive *Salmonella* (pink-red on BG) colonies were stored in glycerol at -80℃ for molecular analysis for confirmation and antibiotic resistance profile.

#### Campylobacter

For *Campylobacter* spp. detection Preston Broth was used for enrichment after filtration and incubated 48 h at 35–37 °C in microaerophilic conditions. Tubes exhibiting growth were plated onto Karmali agar and incubated 48 h at 35–37 °C in microaerophilic conditions. Karmali plates were checked for growth and five presumptive *Campylobacter* spp colonies were stored in glycerol at -80℃ for molecular analysis.

#### Molecular analysis: species confirmation and typing

##### DNA isolation

DNA was isolated from the cells by the boiling method. Isolates were retrieved from glycerol stocks by plating onto specific media (see above) and incubated over night at 37 °C. From each isolate, one colony was picked and added to 500 µL of sterile PCR grade water and incubated 10 min at 95 °C and the boiled cells immediately transferred onto ice for 15 min. Tubes were then centrifuged at 13,000 rpm for 10 min and 400 µl of supernatant was transferred in a clean sterile tube and 3 µL was used as template for PCR.

##### PCR

Species confirmation and typing of the isolates was done by PCR, by using selected primers as shown in Supplementary Table 2. *Vibrio cholerae* was confirmed by PCR based on the *ompW* gene encoding for the outer membrane protein [67]. A multiplex PCR was performed to check if the isolates were *V. cholerae* O1 or O139 and if the isolates possessed the genetic potential of producing cholera toxin [68]. *Campylobacter jejuni* in the isolated *Campylobacter* colonies was confirmed by a PCR assay based on the presence of the gene *hsp60* encoding the heat stable protein as described by Park et al. [69]. *Salmonella* suspected colonies were tested by a PCR as described by Martinez-Ballesteros et al. [70]. A PCR assay was performed as to confirm *E. coli* based on 16 S and *uidA* gene [71].

## Antibiotic resistance profile

Selected cefotaxime resistant *E. coli* and *V. cholerae* isolates were tested for their antimicrobial susceptibilities by replica plating onto Mueller Hinton agar supplemented with antibiotic at breakpoint concentrations (Supplementary Table 3)^72^. The minimal inhibitory concentrations (MIC) considered to represent resistance to a given antibiotic were those determined by CLSI^72^. Isolates with intermediate susceptibility were categorized as being susceptible.

## Physico-chemical parameters

Physico-chemical water quality parameters were measured on site. Conductivity and pH were measured using a PT157 (Palintest, United Kingdom) probe, and temperature was recorded using a PT155 (Palintest, United Kingdom) probe. The Palintest Turbimeter Plus PTH092 was used to analyse the turbidity of the samples. The Palintest Photometer 7100 PTH7100 was used to analyse free and total chlorine. Meters were calibrated on a monthly basis.

### Statistical analysis

Concentrations of *E. coli* (CFU/100 mL) and physico-chemical parameters were logarithmically (base 10) transformed. For *E. coli*, removal was calculated as the difference between the concentrations obtained in the raw water (I) and the concentrations obtained in the treated water (T0). The normality distribution of *E. coli* concentrations and physical-chemical parameters data was checked by Shapiro-Wilk’s test and data analysis and plots were performed using R Studio software *V.* 1.4.1103. The correlations among different parameters using Spearman’s correlation test. Spearman’s coefficient (r) with P values < 0.05 were considered statistically significant.

## Electronic supplementary material

Below is the link to the electronic supplementary material.

Supplementary Material 1

## Data Availability

All data is available in the manuscript and in the supporting material.
