# Supplementary material for: Occurrence of waterborne pathogens and antibiotic resistance in water supply systems in a small town in Mozambique
Source: BMC Microbiol. 2022 Oct 8;22:243. doi: 10.1186/s12866-022-02654-3 (PMC9547466; doi:10.1186/s12866-022-02654-3)
Supplement: Supplementary file 1 — Supplementary Material 1 [file 12866_2022_2654_MOESM1_ESM.docx]

**Supplementary Table 1.** Volumes of water tested per sampling location and microbial parameters**.**

| **Location** | **Volume tested (in mL)** | | | | | |
| --- | --- | --- | --- | --- | --- | --- |
|  | 1. *coli* | *ctx + E.coli* | *Vibrio cholerae* | *Salmonella* | *Campylobacter* | *Total* |
| Inlet | 0.1, 1, 10, 100 | 0.1, 1, 10, 100 | 0.1, 1, 10, 100 | 0.1, 1, 10, 100 | 0.1, 1, 10, 100 | 555.5 |
| Outlet | 100 | 1, 10 and 100 | 10 and 100 | 10 and 100 | 10 and 100 | 541.0 |
| Tap water | 100 | 1, 10 and 100 | 10 and 100 | 10 and 100 | 10 and 100 | 541.0 |

**Supplementary Table 2.** Genetic markers and associated primers used for the molecular confirmation and characterization of the isolates.

| **Organism** | **Primer name** | **Primer sequence** | **Gene** | **Amplicon (bp)** | **Reference** |
| --- | --- | --- | --- | --- | --- |
| *E. coli* | uid-F | CCAAAAGCCAGACAGAGT | *uidA* | 623 | Bej et al., 1991 |
|  | uid-R | GCACAGCACATCAAAGAG |  |  |  |
| *Salmonella* | OMPC-F | ATCGCTGACTTATGCAATCG | *ompC* | 204 | Martinez-Ballesteros et al., 2012 |
|  | OMPC-R | CGGGTTGCGTTATAGGTCTG |  |  |  |
| *V. cholerae* | MER-24 | CACCAAGAAGGTGACTTTATTGTG | *ompW* | 588 | Nandi et al., 2000 |
|  | MER-19 | GAACTTATAACCACCCGCG |  |  |  |
|  | ctxB_F | GGTTGCTTCTCATCATCGAACCAC | *ctxB* | 465 | Hoshino et al., 1998 |
|  | ctxB_R | GATACACATAATAGAATTAAGGAT |  |  |  |
| 1. *jejuni* | campsh_F | CAAGTTGCTACAATCTCAGCCA | *hsp60* | 90 | Park et al., 2011 |
|  | campsh_R | GATAACACCATCTTTGCCCACT |  |  |  |

**Supplementary Table 3.** Antimicrobias used to test resistance of *E. coli* and *V. cholerae* isolates and their breakpoint concentrations

| Antimicrobial Classes | Antimicrobial Agent | Abbrev. | Breakpoint  Concentration (μg/mL) |
| --- | --- | --- | --- |
| β-lactam: Penicillins | Ampicillin | AMP | ≥ 32 |
|  | Amoxicillin-clavulanic ac. | AMC | ≥ 32 |
| β-lactam: Second and Third-generation  Cephalosporins | Cefoxitin | FOX | ≥ 32 |
|  | Cefotaxime | CTX | ≥ 4 |
| Aminoglycosides | Gentamycin | GEN |  |
|  | Streptomycin | STR | ≥ 64 |
|  | Kanamycin | KAN | ≥ 64 |
| Folate Pathway Inhibitors | Trimethoprim-Sulfamethoxazole | SXT | ≥ 4 |
| Tetracyclines | Tetracycline | TET | ≥ 16 |
|  | Doxicycline | DO | ≥ 16 |
| Phenicols | Chloramphenicol | CHL | ≥ 32 |
| Quinolones | Ciprofloxacin | CIP | >4 |
|  | Nalidixic acid | NAL | ≥ 32 |

**Supplementary Table 4.** Mean and standard deviation of free and total chlorine, temperature, turbidity, pH and electric conductivity recorded at each sampling point.

| **Samplingpoint** | **Free Chlorine** | **Total Chlorine** | **Temp** | **Turbidity** | **pH** | **EC** |
| --- | --- | --- | --- | --- | --- | --- |
|  | **mg/l** | **mg/l** | **ºC** | **NTU** |  | **µS/cm** |
| Inlet |  |  | 23.32±  1.66 | 5.27±  3.16 | 8.10±  0.29 | 537.35±  114.49 |
| T0 | 1.19±  0.59 | 1.42±  0.58 | 23.66±  1.54 | 6.99±  3.66 | 8.31±  0.15 | 536.02±  68.13 |
| T1 | 0.49±  0.32 | 0.78±  0.54 | 23.69±  1.59 | 7.84±  5.24 | 8.10±  0.24 | 514.82±  81.58 |
| T2 | 1.07±  0.49 | 1.23±  0.45 | 23.53±  1.64 | 5.97±  3.46 | 8.22±  0.15 | 573.81±  71.38 |
| T3 | 0.96±  0.25 | 1.15±  0.25 | 23.71±  0.62 | 4.54±  1.23 | 8.26±  0.15 | 609.18±  34.31 |
